# Potential Benefits of Glycine, Proline and Hydroxyproline on Growth and Flesh Quality of Mirror Carp (*Cyprinus carpio* var. *specularis*)

**DOI:** 10.3390/ijms26189011

**Published:** 2025-09-16

**Authors:** Rong Zhang, Huijuan Li, Xiaowen Wang, Lili Liu, Hua Zhu

**Affiliations:** Beijing Key Laboratory of Fishery Biotechnology, Fisheries Science Institute, Beijing Academy of Agriculture and Forestry Sciences, Beijing 100097, China; zhangrong@baafs.net.cn (R.Z.); huijuan_li@baafs.net.cn (H.L.); wangxiaowen@baafs.net.cn (X.W.); liulili@baafs.net.cn (L.L.)

**Keywords:** growth, GH/IGF-1, collagen, TGF-β/smad, free amino acids

## Abstract

Collagen content is a key determinant of flesh quality and directly influences consumer acceptance. This study investigated the effects of the main collagen amino acids, glycine, proline and hydroxyproline, on the growth performance and flesh quality of mirror carp. A total of 240 fish (initial weight: 174.4 g) were randomly assigned to a control and three treatment groups, each receiving 5 g of glycine, proline, or hydroxyproline per kilogram of feed for eight weeks. Measured parameters included growth performance, serum hormones, flesh quality traits, free amino acid profiles, and proteomics. Glycine and hydroxyproline increased serum GH and IGF-1 levels (*p* < 0.001), while somatostatin was differentially regulated across treatments. Hydroxyproline notably improved flesh quality by reducing drip loss and increasing muscle moisture and protein content (*p* = 0.022; *p* = 0.040; *p* = 0.026). Collagen levels in muscle and skin were elevated in all treatment groups (*p* < 0.05). Free amino acid analysis revealed elevated levels of leucine and taurine in the glycine group, increased taurine in the proline group, and elevated methionine and taurine in the hydroxyproline group (*p* < 0.05). Proteomic analysis identified differentially expressed proteins enriched in pathways including oxidative phosphorylation, glutathione metabolism, and valine, leucine and isoleucine degradation. These findings suggest that hydroxyproline plays a regulatory role in hormone secretion and muscle quality enhancement, offering new insights for optimizing aquafeed formulations.

## 1. Introduction

In aquaculture, optimizing feed composition aims not only to sustain fish growth but also to improve fish meat quality, directly influences consumer satisfaction and market value [1]. Filet texture is a key determinant of fish flesh quality and directly affects consumer acceptance. Farmed fish typically exhibit a softer texture than wild fish, a trait often perceived as inferior by consumers [2,3,4]. This difference is largely attributed to variations in collagen content, distribution, and cross-linking within the muscle tissue [5,6,7,8]. Therefore, enhancing collagen deposition represents a feasible strategy for improving the texture and overall quality of farmed fish filets [9].

Amino acids, as fundamental components of proteins, are essential not only for protein synthesis but also for regulating various metabolic pathways, including glucose and lipid metabolism, cellular signaling, and stress responses [10]. Beyond supporting growth, amino acids play a crucial role in enhancing product quality, a factor increasingly prioritized by consumers [11].

Glycine, proline, and hydroxyproline, which together constitute 57% of the amino acids in collagen, are essential for its structure and stability, with glycine fitting within the tight triple helix, proline conferring structural rigidity, and hydroxyproline enhancing thermal stability through intramolecular hydrogen bonding [12,13]. Hydroxyproline is a metabolic derivative of proline, catalyzed by prolyl hydroxylase after protein synthesis. It serves not only as a structural component of collagen but also as a substrate in glycine synthesis, potentially conserving proline by reducing its degradation [14,15,16]. Beyond their roles in collagen synthesis, glycine, proline, and hydroxyproline are involved in broader amino acid metabolic networks that may influence meat quality. For example, glycine participates in one-carbon metabolism by donating methyl groups for the remethylation of homocysteine to methionine through the folate cycle [17]. Methionine serves as a precursor for cysteine, glutathione, and taurine, and contributes to the formation of sulfur-containing flavor compounds via strecker degradation [18]. Cysteine and glutathione enhance antioxidant capacity, which plays a crucial role in maintaining meat quality and prolonging shelf life [19].

Research suggests that the endogenous synthesis of glycine and proline may be insufficient to meet the metabolic demands required for optimal growth and health [14,20]. Dietary supplementation with glycine, proline, and hydroxyproline has been proposed to address their limited endogenous production [14,20]; however, the specific metabolic pathways of these amino acids in fish muscle tissue remain poorly understood. This research aims to fill this gap by employing proteomic approaches to elucidate how these amino acids integrate into key metabolic processes in fish muscle. By identifying novel protein expression patterns and metabolic pathways, this research provides novel insights for improving product quality in aquaculture.

Mirror carp (*Cyprinus carpio* var. *specularis*), a freshwater omnivorous fish, is widely cultivated due to its rapid growth, strong disease resistance, high yield, and favorable taste [21]. Given its economic importance and the increasing demand for sustainable protein sources, mirror carp provides an ideal model for investigating the effects of dietary supplements on growth and product quality. Such advancements are essential for increasing aquaculture productivity and improving product quality to meet the growing global demand for sustainable protein sources.

## 2. Results

### 2.1. Growth and Growth Hormones

**Figure 1:**
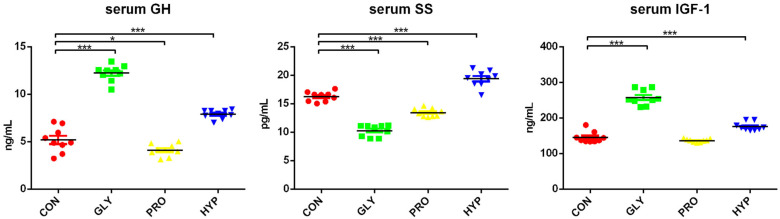
Modulation of serum growth hormone (GH, ng/mL), somatostatin (SS, pg/mL), and insulin-like growth factor-1 (IGF-1, ng/mL) by dietary amino acid supplementation (*n* = 9 per treatment). Significant difference was determined at *p* < 0.05 (*) or *p* < 0.001 (***).

The survival rate of fish was 100% throughout the experiment. No significant differences were observed in the WG, WGR, SGR, or FCR between the control and treatment groups (Table 1). However, notable differences were observed in growth-related hormone levels: both the GLY and HYP treatments significantly increased GH content compared to the control (*p* < 0.001), while PRO supplementation led to a decrease in GH levels (*p* = 0.040). Additionally, when compared with the control, SS levels were reduced in the GLY and PRO groups (*p* < 0.001) but increased in the HYP group (*p* < 0.001), whereas IGF-1 levels were significantly higher in the GLY (*p* < 0.001) and HYP (*p* < 0.001) groups (Figure 1).

### 2.2. Flesh Quality and Nutrient Profiles

**Figure 2:**
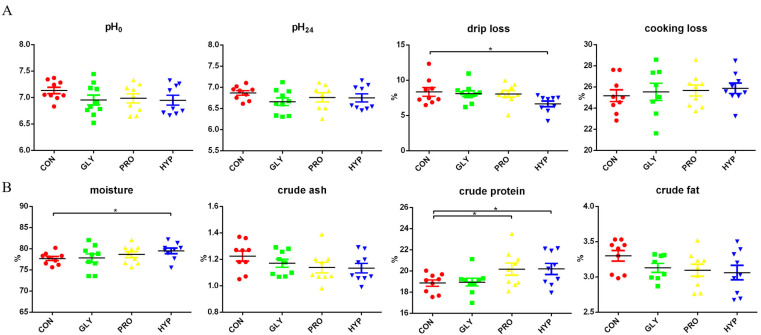
(**A**) Changes in muscle pH, drip loss (%), and cooking loss (%) in response to dietary amino acids. (**B**) Muscle nutrient composition (%) in response to dietary amino acids (*n* = 9 per treatment). Significant difference was determined at *p* < 0.05 (*).

Hydroxyproline supplementation led to significant improvements in fish muscle quality, including a reduction in drip loss (*p* = 0.022) and increases in muscle moisture (*p* = 0.040) and CP content (*p* = 0.026), when compared with the control. Proline supplementation also increased muscle CP content (*p* = 0.035; Figure 2).

### 2.3. Collagen Content

**Figure 3:**
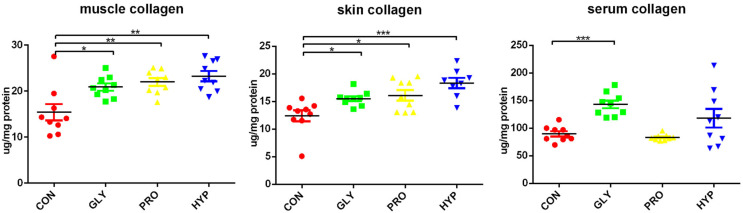
Alterations in collagen contents in skin, muscle, and serum induced by dietary amino acids (*n* = 9 per treatment; μg/mg protein). Significant difference was determined at *p* < 0.05 (*), *p* < 0.01 (**) or *p* < 0.001 (***).

Collagen contents in both muscle and skin were significantly elevated in all treatment groups compared to the control (*p* < 0.05), while serum collagen levels were markedly increased in the GLY group (*p* < 0.001; Figure 3).

### 2.4. Targeted Amino Acids Quantification

**Figure 4:**
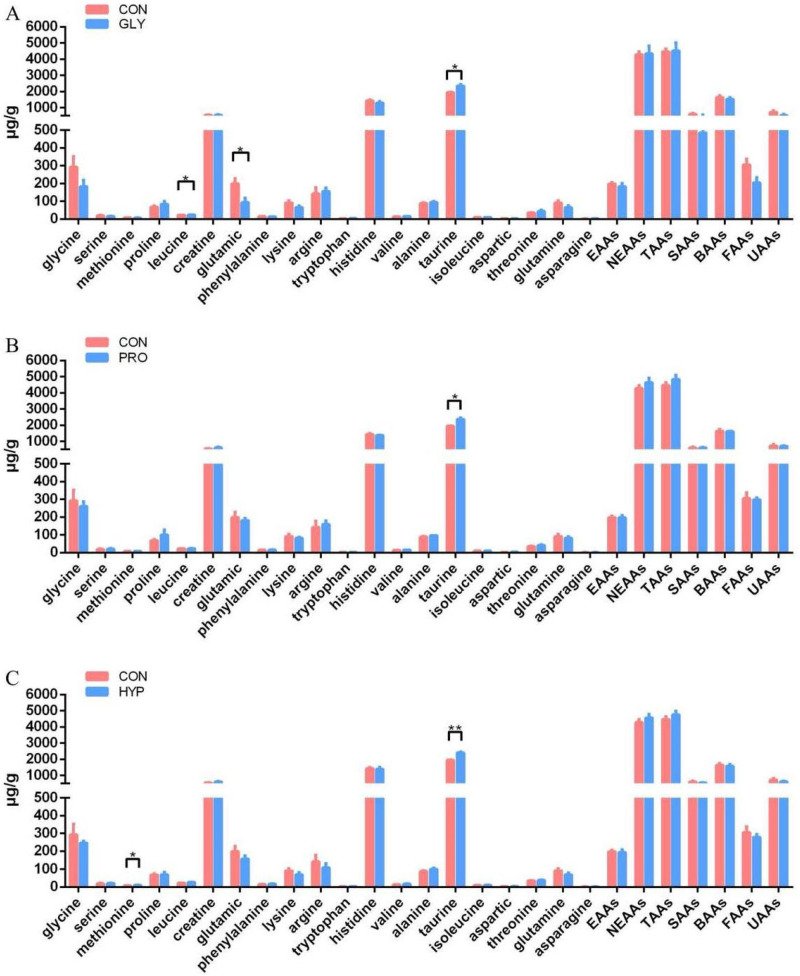
Quantitative profiling of targeted amino acids in muscle tissue between (**A**) CON-GLY, (**B**) CON-PRO, and (**C**) CON-HYP groups (*n* = 3 per treatment, μg/g). EAAs: essential amino acids (sum of Thr, Lys, Met, Val, Leu, Ile, Phe, and Trp); NEAAs: non-essential amino acids (sum of Gly, Ala, Ser, Pro, His, Arg, Glu, Asp, and Tau); TAAs: total amino acids; SAAs: sweet amino acids (sum of Gly, Ala, Ser, Pro, Thr, and Lys); BAAs: bitter amino acids (sum of Met, Val, Leu, Ile, Phe, His, Arg, and Trp); FAAs: flavor amino acids (sum of Asp, Glu, Phe, and Ala); UAAs: umami amino acids (sum of Gly, Ala, Arg, Glu, and Asp). Significant difference was determined at *p* < 0.05 (*), *p* < 0.01 (**).

Targeted amino acids quantification revealed that leucine and taurine levels increased (*p* = 0.048; *p* = 0.017), while glutamic acid decreased (*p* = 0.048), in the GLY treatment. Taurine content was also increased in the PRO treatment (*p* = 0.014). In the HYP treatment, the levels of methionine and taurine were elevated (*p* = 0.022; *p* = 0.002; Figure 4).

### 2.5. Gene Expression

**Figure 5:**
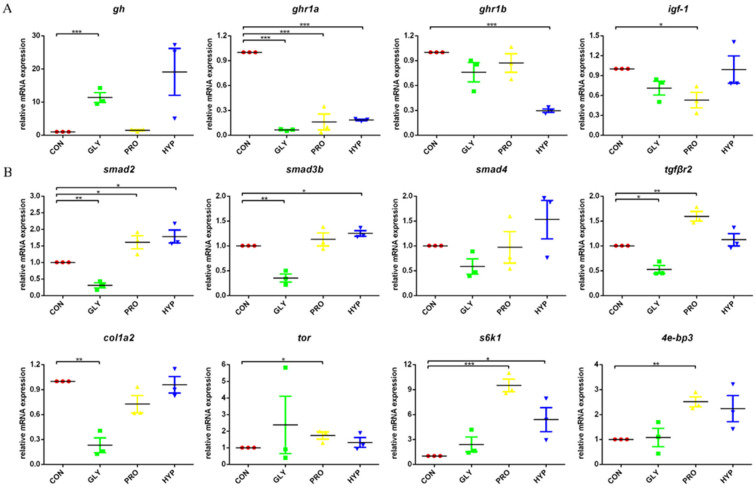
(**A**) Brain expression patterns of gh, ghr1b, ghr1a, and igf-1 in response to dietary amino acids. (**B**) Muscle expression patterns of smad2, smad3b, smad4, tgfβr2, col1a2, tor, s6k1, and 4e-bp3 in response to dietary amino acids (*n* = 3 per treatment). Significant difference was determined at *p* < 0.05 (*), *p* < 0.01 (**) or *p* < 0.001 (***).

In brain tissue, the expression of gh was significantly up-regulated in the GLY group (*p* < 0.001), while the expression of ghr1a was down-regulated (*p* < 0.001). Proline supplementation down-regulated the expression of ghr1a and igf-1 (*p* = 0.001; *p* = 0.016), while hydroxyproline supplementation reduced the expression of both ghr1a and ghr1b (*p* < 0.001; *p* < 0.001; Figure 5A).

In muscle tissue, glycine supplementation led to the significant down-regulation of genes associated with collagen synthesis and growth factor signaling, including smad2, smad3b, tgfβr2, and col1a2 (*p* = 0.001; *p* = 0.001; *p* = 0.013; *p* = 0.001). Conversely, proline supplementation up-regulated the expression of smad2, tgfβr2, tor, s6k1, and 4e-bp3 (*p* = 0.035; *p* = 0.003; *p* = 0.030; *p* < 0.0001; *p* = 0.002). Furthermore, hydroxyproline supplementation up-regulated the expression of smad2, smad3b, and s6k1 (*p* = 0.016; *p* = 0.012; *p* = 0.039; Figure 5B).

### 2.6. Proteomics Analysis

**Figure 6:**
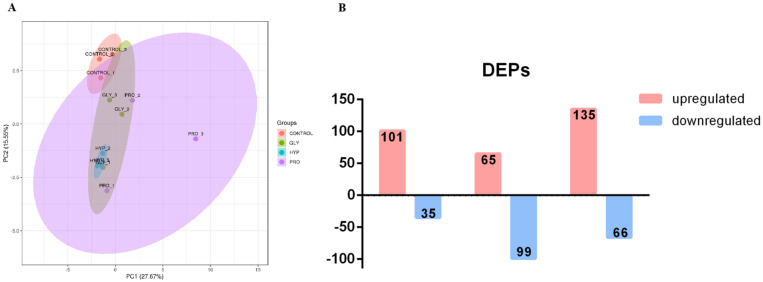
(**A**) Principal component analysis (PCA) showing protein expression variance between treatment and control groups. (**B**) The number of up-regulated (fold change > 1.2) and down-regulated proteins (fold change < 0.83, number of proteins).

**Figure 7:**
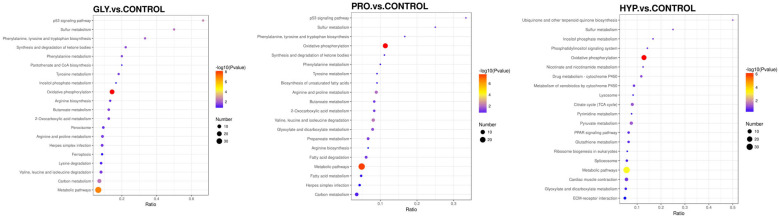
KEGG pathway enrichment of upregulated proteins between the treatment group and the control group (*n* = 3 per treatment).

Principle component analysis (PCA) depicted distinct separation among treatments (Figure 6A). A total of 136 DEPs were detected in the GLY treatment, 164 DEPs in the PRO treatment, and 201 DEPs in the HYP treatment compared to the control (Figure 6B). The enriched KEGG pathways for upregulated DEPs are presented in Figure 7. Compared with the control group, upregulated proteins in the GLY group were mainly enriched in pathways such as oxidative phosphorylation, metabolic pathways, and carbon metabolism. In the PRO group, upregulated proteins were primarily enriched in oxidative phosphorylation, metabolic pathways, and valine, leucine and isoleucine degradation. The HYP group showed enrichment in oxidative phosphorylation, metabolic pathways, and ECM–receptor interaction.

**Figure 8:**
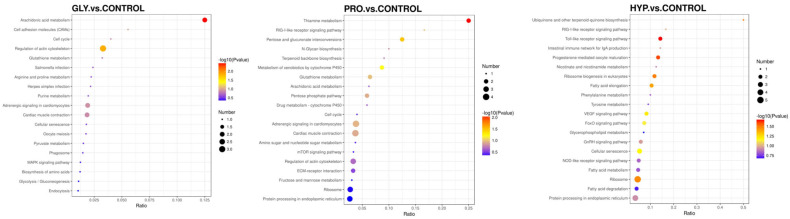
KEGG pathway enrichment for downregulated proteins between the treatment group and the control group (n = 3 per treatment).

The enriched KEGG pathways for downregulated DEPs are presented in Figure 8. Compared with the control group, the downregulated proteins in the GLY group were mainly enriched in arachidonic acid metabolism, the regulation of actin cytoskeleton, and adrenergic signaling in cardiomyocytes. In the PRO group, the downregulated proteins were primarily enriched in thiamine metabolism, mTOR signaling pathway, ECM–receptor interaction, and protein processing in the endoplasmic reticulum. In the HYP group, they were mainly enriched in the Toll-like receptor signaling pathway and protein processing in the endoplasmic reticulum.

**Figure 9:**
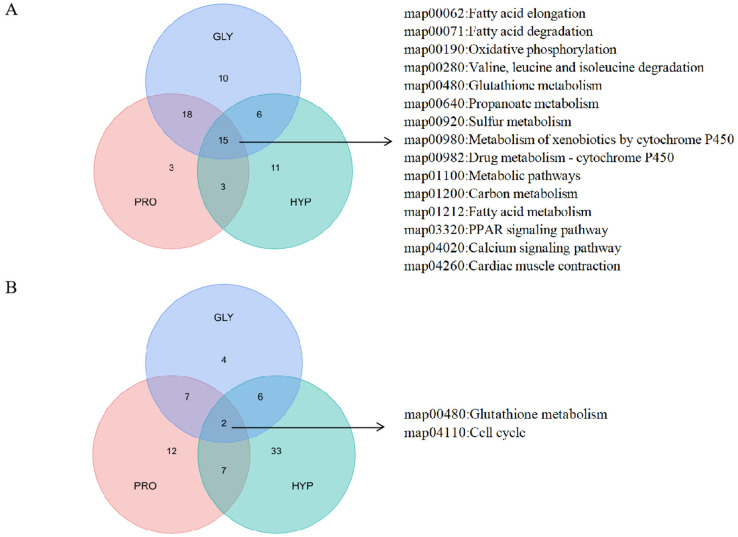
Venn diagrams showing the number of shared and distinct KEGG pathways enriched among (**A**) upregulated and (**B**) downregulated proteins (n = 3 per treatment).

**Figure 10:**
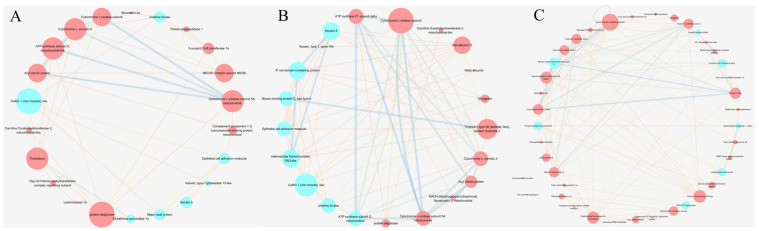
Protein–protein interaction (PPI) networks of differentially expressed proteins between the treatment group and the control group (n = 3 per treatment). (**A**) GLY; (**B**) PRO; (**C**) HYP. Red indicates up-regulated expression, while blue indicates down-regulated expression. Blue lines denote gene co-occurrence evidence, and yellow lines denote text-mining evidence of functional association.

**Figure 11:**
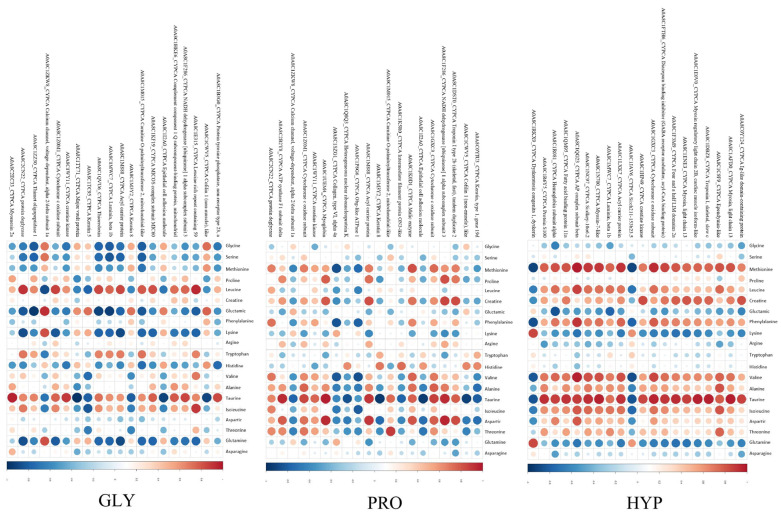
Correlation matrix depicting relationships between top 20 differentially expressed proteins and 20 targeted amino acids. Red and blue indicate positive and negative correlations, respectively; color intensity denotes correlation strength. Circle size reflects correlation magnitude (ρ).

Among the upregulated DEPs, 15 KEGG pathways were commonly enriched across all treatments, including oxidative phosphorylation, valine, leucine and isoleucine degradation, and glutathione metabolism (Figure 9A). Conversely, glutathione metabolism and cell cycle pathways were commonly enriched among downregulated DEPs in all treatments (Figure 9B). The PPI network of differentially expressed proteins (DEPs) revealed key proteins involved in collagen metabolism across different group comparisons. Thioredoxin, protein deglycase, and cofilin 1 (non-muscle)-like proteins were prominent between the GLY and CON groups. Cofilin 1(non-muscle)-like, epithelial cell adhesion molecular, and keratin 8 were significant between the PRO and CON groups. Heat shock protein beta-1, protein disulfide-isomerase A6 (PDI A6), and peptidylprolyl isomerase Ab (Cyclophilin) were key proteins distinguishing the HYP and CON groups (Figure 10). Furthermore, correlations between protein expression and amino acid quantification are presented in Figure 11.

## 3. Discussion

Glycine, traditionally considered a non-essential amino acid in aquatic species, may limit maximal growth due to insufficient de novo synthesis from serine, choline, threonine, and 4-hydroxyproline [22]. Glycine plays a crucial role in regulating hypothalamic-pituitary function and GH release in humans [23]. In our study, dietary glycine supplementation increased serum levels of GH and IGF-1, while reducing SS levels. IGF-1 is a key regulator of muscle anabolism, activating protein synthesis pathways [24]. However, the effects of glycine on growth promotion in aquatic species remain inconclusive, with some studies reporting positive outcomes in species like Pacific white shrimp, *Litopenaeus vannamei,* and Nile tilapia, *Oreochromis niloticus* [25,26], while others have shown no significant effects in grass carp, *Ctenopharyngodon idella* [27]. Similarly, research indicates that endogenous synthesis proline may not be sufficient to meet the metabolic demands necessary for optimal growth and health [14]. Dietary proline has been found to improve growth in large yellow croaker, *Larimichthys crocea* [28], but had no significant impact on juvenile turbot, *Scophthalmus maximus* L. [29]. Hydroxyproline is the only free amino acid in tissues that shows a positive correlation with the growth rate of juvenile salmon, *Salmo salar* L. [30]. It has shown growth-promoting effects in Atlantic salmon, *Salmo salar* L. [31], turbot, *Scophthalmus maximus* L. [32], large yellow croaker, *Larimichthys crocea* [33], and Chinese perch, *Siniperca chuatsi* [9]. However, inconsistent outcomes have been reported in juvenile turbot, *Scophthalmus maximus* L. [34], and yellow river carp, *Cyprinus carpio haematopterus* [35].

These variations may be attributed to differences in nutrient requirements between omnivorous and carnivorous fish species. Moreover, plant protein sources generally have lower concentrations of glycine, proline, and hydroxyproline compared to fish meal [32,36,37]. The inclusion rate of fish meal in the basal diet may thus influence the growth-promoting effects of these amino acids. Our results suggested that dietary supplementation with GLY and HYP increased GH and IGF-1 levels but reduced brain ghr1a or ghr1b expression, suggesting GH receptor desensitization due to prolonged amino acid exposure. This may explain the lack of growth performance response to elevated hormone levels. Additionally, the limited number of cage replicates may have reduced the statistical power to detect smaller treatment effects, and future studies should consider increasing replication to enhance the robustness of the results.

pH and water-holding capacity are important parameters for assessing flesh quality [38,39]. Post-mortem glycolysis of muscle glycogen produces lactic acid, leading to a drop in pH [40]. Rapid pH declines are linked to reduced water-holding capacity in farmed Atlantic cod, *Gadus morhua*, compromising flesh quality [41,42]. Dietary supplementation with glycine, proline, and hydroxyproline did not significantly affect muscle pH, likely because these amino acids primarily facilitate protein synthesis rather than directly influencing glycolysis. Our results also demonstrated that dietary hydroxyproline improved flesh quality in terms of drip loss, probably due to the hydroxyl groups in collagen binding water molecules to prevent moisture loss [43].

Flesh texture is strongly associated with collagen content, distribution, and cross-linking [44]. Collagen, rich in glycine, proline, and hydroxyproline, forms a triple-helical structure that imparts stability and tensile strength [14,45]. Dietary glycine has been shown to enhance collagen deposition in broilers [46,47], while proline increases collagen in juvenile turbot muscle [29], salmon skeleton [31], and the swim bladder of Chu’s croaker, *Nibea coibor* [48]. Insufficient endogenous synthesis of these amino acids may limit collagen production and growth in fish [32]. Glycine, proline, and hydroxyproline, which together constitute 57% of the amino acids in collagen, are essential for its structure and stability, with glycine fitting within the tight triple helix, proline conferring structural rigidity, and hydroxyproline enhancing thermal stability through intramolecular hydrogen bonding [12,13]. Dietary hydroxyproline increases total collagen content in tissues, and improves muscle hardness, springiness, and chewiness in fish [32,33,34,35]. Our results showed that increasing the dietary ratios of glycine to lysine (0.76 to 0.98%), proline to lysine (0.836 to 1.06%), and hydroxyproline to lysine (0.122 to 0.34%) significantly enhanced collagen content in fish flesh. Thus, to improve meat quality in mirror carp, particularly when substituting fishmeal with plant-based ingredients, it is recommended to maintain a minimum threshold of these three amino acids in feed formulations. Ingredients rich in glycine, proline, and hydroxyproline, such as hydrolyzed feather meal [14], can serve as effective alternatives to balance amino acid profiles. However, the absence of these amino acid gradients in our study limited the determination of the optimal range.

Additionally, glycine, proline, and hydroxyproline are broadly involved in amino acid metabolic networks. Our results showed that taurine content in muscle was significantly increased in all three treatment groups. Taurine is abundant in fish muscle and plays important roles in bile acid conjugation, lipid absorption, calcium regulation, and muscle contraction [49,50,51,52,53], which is consistent with our KEGG enrichment results. The potential metabolic pathway may involve glycine contributing to one-carbon metabolism by donating methyl groups for the remethylation of homocysteine to methionine [17], with methionine subsequently serving as a precursor for taurine. Furthermore, in the glycine group, leucine content was significantly increased. Leucine can be further metabolized to acetyl–CoA and succinyl–CoA, which enter the TCA cycle to support ATP production [54]. It has also been shown to increase mitochondrial content and oxygen consumption, and to upregulate genes related to mitochondrial biogenesis [55]. As previously described, the interconversion among glycine, proline, and hydroxyproline suggests the presence of a reversible amino acid pool closely linked to collagen turnover [14,15,16]. Consistent with this, our study revealed that differentially expressed proteins (DEPs) in all three treatment groups, when compared with the control, were frequently enriched in KEGG pathways such as oxidative phosphorylation, valine, leucine and isoleucine degradation, and glutathione metabolism. To our knowledge, the effects of glycine, proline, and hydroxyproline on the fish muscle proteome have not been previously reported. Our findings address this gap to elucidate the integration of these amino acids into key metabolic pathways in fish muscle.

The transforming growth factor β (TGF-β)/Smads signaling pathway plays a crucial role in regulating collagen gene expression [56,57]. Dietary proline has been found to upregulate genes associated with collagen synthesis (col1a1, col1a2, and ctgf) while down-regulating TIMP2 [48]. Additionally, the TGF-β/Smads pathway is not the only regulator of collagen synthesis. In mammals, collagen synthesis is regulated by intricate signaling pathways, including TGF-β/Smads, phosphatidylinositol-3-kinase/serine/threoninekinase (PI3K/Akt), mitogen-activated protein kinase (MAPK), and Wnt/β-catenin [58,59,60]. The mammalian target of rapamycin (mTOR) actively participates in regulating cell growth, proliferation, and differentiation, positively influencing collagen production in human fibroblasts [61]. Our data suggest that the collagen-enhancing effects may be mediated via the TGF-β/Smads and TOR pathway. Hug proteins such as protein disulfide-isomerase A6 and heat shock protein beta-1 have been implicated in regulating TGF-β signaling, influencing Smad3 activation, epithelial-to-mesenchymal transition, and fibrogenic responses [62,63]. Additionally, peptidylprolyl isomerase Ab (Cyclophilin A) modulates mTOR signaling, impacting cell proliferation and metabolism [64].

## 4. Materials and Methods

### 4.1. Experimental Design

The experiment was conducted at a commercial aquaculture farm located in Beijing, China, from July to September. Following a 2-week acclimation period, 240 fish (initial weight: 174.4 g) were randomly allocated to one control group and three treatment groups, each supplemented with 0.5% glycine (GLY), 0.5% proline (PRO), and 0.5% hydroxyproline (HYP). Each treatment comprised three replicate cages, with 20 fish in each replicate. The fish were reared in a pond culture system, with three fish cages per treatment, each measuring 3 m × 3 m × 1.2 m. The experiment duration spanned 8 weeks, during which the initial stocking density averaged 0.32 kg/m^3^, increasing to 0.55 kg/m^3^ by the end of the study. Fish were fed twice a day, with the feeding rate adjusted to 2% of the initial body weight to ensure all feed was consumed within 5 min. Ingredient compositions and nutritional profiles of the feed are presented in Tables S1 and S2, respectively.

### 4.2. Sampling

All fish were weighed by replicate both at the initiation and termination of the 8-week rearing phase. At the end of the experiment, nine fish per treatment (three from each replicate) were randomly chosen and received an anesthesia overdose, administered as 50 mg/L of tricaine methane–sulfonate (MS-222), to ensure fish did not regain consciousness at any stage, particularly during dissection. Blood samples were drawn from the caudal vein and subsequently centrifuged at 10,000× *g* for 10 min at 4 °C. Serum was collected for the assessment of growth hormone (GH), somatostatin (SS), insulin-like growth factor-1 (IGF-1), and hydroxyproline concentrations. Then, the fish were positioned on a cooling plate, and skin samples were obtained for hydroxyproline content analysis. Muscle samples were removed from the region located behind the operculum and above the lateral line on the left side of each fish, then sectioned consecutively for analysis of flesh quality, collagen content, and LC−MS/MS proteomics, as well as undergoing a targeted amino acids quantification and qRT-PCR. Corresponding muscle samples from the right side of each fish were collected for nutrient profile analysis. Finally, whole brain tissue was collected for subsequent qRT-PCR analysis. The experimental protocol was approved by the Animal Ethics Committee of the Beijing Academy of Agriculture and Forestry Sciences (BAAFS-KJLL-20240801, Beijing, China), ensuring strict compliance with ethical guidelines and promoting the humane treatment of animals. Every effort was made to minimize fish discomfort and distress throughout the course of the experiments.

### 4.3. Measurements

#### 4.3.1. Growth and Growth Hormones

Weight gain (WG, g) = final body weight − initial body weight

Weight gain rate (WGR, %) = 100 × (final body weight- initial body weight)/initial body weight

Specific growth rate (SGR, %) = 100 × [ln (final body weight) − ln (initial body weight)]/days

Feed conversion rate (FCR, %) = 100 × feed intake/(final body weight − initial body weight)

Concentrations of growth hormone (GH), somatostatin (SS) and insulin-like growth factor-1 (IGF-1) were assessed using the enzyme-linked immunosorbent assay (ELISA) method with commercially available kits produced from the Beijing Sino-uk Institute of Biological Technology, Beijing, China.

#### 4.3.2. Flesh Quality, Nutrient Profiles and Collagen Content

Muscle pH was recorded at 0 h and 24 h post-slaughter with a calibrated pH probe (Testo 205 pH meter, Testo AG, Lenzkirch, Germany). Drip loss was quantified by subjecting muscle samples to centrifugation at 500× *g* for 10 min [65]. Muscle samples were weighed before centrifugation (M1) and after centrifugation (M2). Cooking loss was determined by heating muscle samples in a water bath at 70 °C for 20 min. Muscle samples were weighed before cooking (M3) and after cooling (M4) [66].

drip loss (%) = (M1 − M2)/M1 × 100.

cooking loss (%) = (M3 − M4)/M3 × 100.

In accordance with the methods of AOAC (2005), moisture content was assessed by drying samples in an oven set to 105 °C; crude ash (Ash) content was measured via combustion in a muffle furnace at 550 °C, crude protein (CP) content was analyzed using the Kjeldahl method; and crude fat content was measured by the Soxhlet ether extraction procedure [67]. The hydroxyproline contents of muscle, skin, and serum were quantified using the chloramine T method [68]. Collagen content was estimated by multiplying hydroxyproline levels by 8, assuming that hydroxyproline constitutes 12.5% of collagen in connective tissue [67].

#### 4.3.3. Targeted Amino Acid Quantification

Muscle samples were homogenized in acetonitrile/methanol (1:1) with internal standards, centrifuged, and analyzed by UHPLC-MS/MS (ExionLC™ AD UHPLC-QTRAP^®^ 6500+, AB Sciex, Framingham, MA, USA). The operation parameters were as follows: ACQUITY UPLC BEH Amide column (2.1 × 100 mm, 1.7 μm); temperature, 50 °C; mobile phase A, 0.1% formic acid in 5 mM Ammonium acetate; mobile phase B, 0.1% formic acid in acetonitrile; injection volume, 1.0 μL; flow rate, 0.3 mL/min. The gradient was as follows: 80% B for 0.5 min, 80–70% B over 2 min, 70–45% B for 4 min, and 45–80% B by 6.01 min, before being held at 80% B until 9 min.

Mass spectrometer was operated in positive multiple-reaction monitoring (MRM) mode under the following conditions: IonSpray voltage, 5500 V; curtain gas, 35 psi; Ion Source Temperature, 550 °C; gas 1, 50 psi; gas 2, 60 psi. Standard curves of 20 amino acids were used to quantify free amino acids [69].

#### 4.3.4. Quantitative Real-Time Polymerase Chain Reaction (qRT-PCR)

Total RNA was extracted using the EASYspin Plus Rapid Tissue RNA Extraction Kit (RA28, AIBOSEN BIO, Beijing, China). RNA from three fish per replicate was pooled. cDNA was synthesized using the TRUEscript RT MasterMix (PR5802, AIBOSEN BIO, Beijing, China). For qRT-PCR, 2 × SYBR Green qPCR Mix (PR3302, AIBOSEN BIO, Beijing, China) was used. A 25 μL reaction mixture was prepared to assess the mRNA expression. For muscle tissue, a mixture consisted of 12.5 µL of 2 × SYBR qPCR Mix, 0.5 µL each of forward and reverse primers, 2 µL cDNA, and 9.5 µL RNase-free ddH2O. For brain tissue, a mixture contained 12.5 µL of SYBR qPCR Mix, 0.5 µL of each primer, 1 µL cDNA, and 10.5 µL ddH2O. The PCR conditions included an initial step at 95 °C for 2 min, followed by 40 cycles of 95 °C for 15 s, 60 °C for 30 s (20 s for brain), and 72 °C for 30 s. All assays were performed in triplicate, and the relative mRNA expression was calculated using the 2^−ΔΔCT^ method. Primer sequences are listed in Table S3.

#### 4.3.5. Total Protein Extraction, TMT Labeling, and LC−MS/MS Proteomics Analysis

Muscle tissue was homogenized in liquid nitrogen, and equal amounts from three fish per replicate were pooled. Samples were lysed, sonicated on ice for 5 min, and centrifuged at 12,000× *g* for 15 min at 4 °C. The supernatant was reduced with 10 mM DTT at 56 °C for 1 h, alkylated with iodoacetamide for 1 h in the dark, and precipitated with acetone at −20 °C for 2 h. After centrifugation and rinsing, the pellet was dissolved in 100 µL buffer (8 M Urea, 100 mM TEAB, pH 8.5) [70].

Protein digestion was performed with 1.5 µg of trypsin in 100 mM TEAB at 37 °C for 4 h, followed by overnight digestion with additional trypsin and calcium chloride. The pH was adjusted to <3, and the sample was desalted on a C18 column. The eluate was lyophilized, dissolved in 100 μL 0.1 M TEAB, and labeled with TMT reagent for 2 h. The reaction was terminated with 8% ammonium hydroxide, then samples were mixed, desalted, and lyophilized [71].

Separation was performed using a Waters BEH C18 column on an EASY-nLCTM 1200UHPLC system (Thermo Fisher Scientific, Waltham, MA, USA). Mass spectrometry was performed on a Q Exactive™ system (Thermo Fisher) in DDA mode with the following parameters: ion spray voltage of 2.3 kV, ion transport tube temperature of 320 °C, full scan range of 350–1500 *m*/*z*, resolution of 60,000 (at 200 *m*/*z*), automatic gain control (AGC) target of 3 × 106, and maximum ion injection time of 20 ms. The top 40 precursors were fragmented by higher-energy collisional dissociation (HCD) and analyzed in MS/MS.

Spectra were matched to a protein database (1865378-uniprotkb_taxonomy_id_7962_2023_07_21.fasta) using Proteome Discoverer 2.5 (PD2.5, Thermo) with mass tolerances of 10 ppm for precursor ions and 0.02 Da for fragments. Differentially expressed proteins (DEPs) were detected with *p* < 0.05 and fold changes >1.2 or <0.83. The databases of the Kyoto encyclopedia of genes and genomes (KEGG) were analyzed [72], and STRING DB was used to predict protein–protein interaction (PPI) networks (http://STRING.embl.de/) (accessed on 9 October 2024) [73].

### 4.4. Statistical Analysis

The data for growth, serum parameters, flesh quality, nutrient profiles, qRT-PCR, and targeted amino acid quantification were assessed using one-way analysis of variance (SAS Institute Inc., Cary, NC, USA). *p* < 0.05 denoted statistical difference.

## 5. Conclusions

Integrating nutritional thresholds of glycine, proline, and hydroxyproline into feed formulation has the potential to improve flesh quality in mirror carp, providing a promising strategy for developing optimal amino acid models to support high-quality aquaculture.

## Figures and Tables

**Table 1:** Effect of dietary amino acids on growth performance of fish (*n* = 3 per treatment).

Items	CON	GLY	PRO	HYP	*p*-Value
Survival rate (%)	100	100	100	100	-
Initial weight (g)	175.00 ± 2.50	177.50 ± 11.46	167.50 ± 2.50	177.50 ± 11.45	0.450
Final weight (g)	309.83 ± 17.85	315.52 ± 20.27	299.07 ± 5.54	295.23 ± 17.65	0.446
Weight gain (g)	134.83 ± 15.37	138.02 ± 16.09	131.58 ± 5.47	117.73 ± 14.99	0.346
Weight gain rate (%)	76.97 ± 7.67	77.97 ± 10.27	78.57 ± 3.66	66.54 ± 9.77	0.307
Specific growth rate (%)	1.32 ± 0.10	1.34 ± 0.14	1.35 ± 0.05	1.18 ± 0.14	0.296
Feed conversion rate	0.88 ± 0.10	0.87 ± 0.11	0.90 ± 0.04	1.01 ± 0.12	0.295

## Data Availability

The data used to support the findings of this study are available from the corresponding author upon request.

## Supplementary Materials

The following supporting information can be downloaded at: https://www.mdpi.com/article/10.3390/ijms26189011/s1. References [74,75] are cited in Supplementary Materials File.
