# Supplementary material for: Potential Benefits of Glycine, Proline and Hydroxyproline on Growth and Flesh Quality of Mirror Carp (Cyprinus carpio var. specularis)
Source: Int J Mol Sci. 2025 Sep 16;26(18):9011. doi: 10.3390/ijms26189011 (PMC12470109; doi:10.3390/ijms26189011)
Supplement: Supplementary file 1 [file ijms-26-09011-s001.zip › ijms-3814449-supplementary.pdf]

International Journal of Molecular Sciences

MDPI

Table S1. Ingredient compositions of the extruded feed.

Ingredients	CON	GLY	PRO	HYP
soybean meal	35.0	35.0	35.0	35.0
fish meal	20.0	20.0	20.0	20.0
wheat flour	18.2	18.2	18.2	18.2
wheat bran	20.0	20.0	20.0	20.0
fish oil	2.8	2.8	2.8	2.8
1% Vitamin premix[1]	1.0	1.0	1.0	1.0
1% Trace mineral premix[2]	1.0	1.0	1.0	1.0
zeolite	2.0	1.5	1.5	1.5
crystalline glycine	0.0	0.5	0.0	0.0
crystalline proline	0.0	0.0	0.5	0.0
crystalline hydroxyproline	0.0	0.0	0.0	0.5

[1] 1% vitamin premix provided: vitamin A 8,800 IU/kg, vitamin D 1,670 IU/kg, vitamin E 120 IU/kg, vitamin B1 5.2 mg/kg, vitamin B2 16.5 mg/kg, vitamin B6 17.6 mg/kg, vitamin K 10.6 mg/kg, folic acid 5.8 mg/kg, biotin 0.68 mg, niacin 70.8 mg/kg, D-pantothenicacid 47.3 mg/kg.

[2] 1% trace mineral premix provided: Cu 6.6 mg/kg, Fe 200 mg/kg, Zn 110 mg/kg, Mn 38.2 mg/kg, I 1.21 mg/kg, and Se 0.25 mg/kg.

Table S2. Nutrient profiles of the control feed.

Nutrients	%
dry matter[1]	89.65
crude protein[1]	35.02
crude fat[1]	6.04
ash[1]	10.01
neutral detergent fiber[1]	10.11
acid detergent fiber[1]	4.12
lysine[1]	2.26
methione[1]	0.68
threonine[1]	1.32
tryptophan[1]	0.45
glycine[2]	1.72
proline[2]	1.89
hydroxyproline[2]	0.276

[1] Data are measured according to AOAC (2005). [2] Data are calculated based on the measured values of individual ingredients.

Table S3. Primer sequences.

Gene[1]	Primer sequences (5'-3')	Length (pb)	Tm (°C)	Reference	R^2
β-actin	F: CCTGTATGCCAACAC-CGTGCTG	22	61.47	Fazelan et al. (2020) [74]	0.946
	R: CTTCATGGTGGAGGGAG-CAAGG	22	60.6		
smad2	F: GGTGGAGACGCCAG-TTCTTC	20	57.56	XM_042764688.1	0.991
	R: GGGAA-GCTGGTGTTTTCAGG	20	55.89		
smad3b	F: CCCTTACCACTAC-CAGCGTG	20	57.51	XM_019122262.2	0.941

	R: AAAGGTTTGGG-GAGCCTGTG	20	56.7		
smad4	F: CCCCTGGGATTGTGG-GATTC	20	55.65	XM_042772638.1	
	R: TGAGGCGATCTG-CAACAGTC	20	58.77		
tgf-βr2	F: TCAAAAGTCTGCCCGAG-TCC	20	57.27	XM_042776165.1	0.996
	R: CGGAGGTTACTCCCGTTCAC	20	56.88		
col1a2	F: TCCTGATGGAAACAAGGGA-GAG	22	57.47	XM_042776972.1	0.991
	R: GCTACCACGAGGAC-CAGCA	19	58.67		
tor	F: GCAGCAGTTTGTGAGTGGC	19	57.23	FJ899680.1	0.996
	R: GCAGGTAGTTGG-CAAAACGG	20	57.10		
4e-bp3	F: CAGGGGGAACTCG-GATCATT	20	56.32	XM_042770682.1	0.995
	R: ATGGTGTGAAGGAAC-GGTCA	20	55.24		
s6k1	GGTGCATGTCACCTTATGGG	20	55.01	XM_042771062.1	0.998
	AGCTGGCAGCACTTCTAGTC	20	59.07		
gh	F: TGGTCGTAC-CAGAAGGTGTG	20	55.88	Khorshidi et al. (2022) [75]	0.990
	R: TCTGAGGTT-GTTCTCCCCCA	20	56.27		
ghr1a	F: GATCTGGACCTCGTACTG-CATTC	25	59.03	XM_042729945.1	0.953
	R: ACTCTCCAGGTCCAG-CATTTCC	24	59.09		
ghr1b	F: GGGCTGAACTGGACTC-TACTGAA	25	59.47	XM_042762469.1	0.993
	R: GCGCACCCGGACTTCAT-ACT	22	59.64		
igf-1	F: GCCCAAGGACAGCAAA-GAAACC	24	58.95	XM_042740812.1	0.972
	R: TTTCTTCCCCCTGTGTTTCCTC	24	56.50		

[1]*smad2*: mothers against decapentaplegic homolog2; *smad3b*: mothers against decapentaplegic homolog 3b; *smad4*: mothers against decapentaplegic homolog 4; *tgf-βr2*: TGF-beta receptor type-2; *col1a2*: collagen type I alpha 2; *tor*: target of rapamycin; *4e-bp3*: 4E binding protein 3; *s6k1*: S6 kinase1; *gh*: growth hormone; *igf-1*: insulin-like growth factor -1; *ghr1a*: growth hormone receptor 1a; *ghr1b*: growth hormone receptor 1b.
